# Online Positive Interventions to Promote Well-being and Resilience in the Adolescent Population: A Narrative Review

**DOI:** 10.3389/fpsyt.2017.00010

**Published:** 2017-01-30

**Authors:** Rosa M. Baños, Ernestina Etchemendy, Adriana Mira, Giuseppe Riva, Andrea Gaggioli, Cristina Botella

**Affiliations:** ^1^CiberObn ISCIII, Valencia, Spain; ^2^Red de Excelencia PROMOSAM (PSI2014-56303-REDT), Madrid, Spain; ^3^Universidad de Valencia, Valencia, Spain; ^4^University of Zaragoza, Teruel, Spain; ^5^Universitat Jaume I, Castelló, Spain; ^6^Interactive Communication and Ergonomics of NEw Technologies – ICE-NET Lab, Milano, Italy; ^7^Applied Technology for Neuro-Psychology Lab, Milano, Italy

**Keywords:** adolescents, mental health, prevention, positive psychology, online interventions, Internet

## Abstract

Numerous studies have shown an alarming prevalence of depression, anxiety, and behavior disorders in youth. Thus, prevention of psychological problems in this population becomes crucial. According to the World Health Organization (1), prevention should also include the promotion and development of the individual’s strengths in order to reduce vulnerability to suffering from mental disorders. In addition, other key elements of prevention are the reach, adoption, implementation, and maintenance of interventions. The information and communication technologies, especially the Internet, have much to offer in terms of the prevention and promotion of positive mental health in adolescents. This paper reviews these fields of research—prevention, positive psychology, Internet, and adolescents—and discusses the potential of positive interventions delivered over the Internet as effective and sustainable health promotion tools. The paper provides a brief description of the systems developed so far and a summary of selected features of the studies detected in the literature review. The overall conclusions are that there is a need for more controlled studies with long-term follow-ups, the interventions should be designed considering the specific features of the target users and the specific contexts where the interventions will be delivered, and they could be enhanced by the use of other technologies, such as smartphones, sensors, or social networks.

## Introduction

Mental health disorders represent a large proportion of the disease burden (2), decreasing quality of life, and increasing the vulnerability to developing severe disabling diseases. Moreover, they have an important economic impact on society (career, family cost, low productivity, etc.) (3, 4). For the adolescent population, these problems take on special importance. Numerous studies have shown an alarming prevalence of depression, anxiety, and behavior disorders in youth (5, 6), and both retrospective and prospective research has shown that most adulthood psychiatric disorders begin in childhood and adolescence (7–10).

Estimates indicate that about 10–20% of children and adolescents suffer from mental health problems worldwide, but large differences in prevalence estimates across countries have also been noted (1). Thus, prevention of psychological problems in the youth population is crucial, and the promotion of young people’s mental health and well-being is considered a key priority in the *EU Framework for Action* and *H2020* (11). Evidence shows that mental illness can be prevented in both adult and youth populations (12–15), and that intervening during childhood and adolescence maximizes the benefits of prevention tasks (16, 17).

According to the World Health Organization (1), prevention involves different actions aimed to reduce risk factors, interrupt the disease’s progress, and reduce its consequences. However, mental health is more than just the absence of mental illness; instead, it is “*a state of well-being in which the individual realizes his or her own abilities, can cope with the normal stresses of life, can work productively, and is able to make a contribution to his or her community* …” [(18), p.1]. Mental health also implies a positive emotional state, positive expectations for the future, and an adaptive way of interpreting reality (19). Therefore, prevention should also include the promotion and development of the individual’s strengths, encouraging protective variables that empower the person and act as barriers and shields that can reduce vulnerability to suffering from mental disorders (1).

Evidence points out that some human strengths act as buffers against mental illness (20). Nevertheless, literature on adolescents and a prevention approach addressed to fostering individual strengths and protective variables, such as resilience, optimism, and positive emotions, is scarce (21). Although support for interventions designed to enhance these variables in adolescence is steadily growing, more research is needed (22).

Another key element of prevention involves reaching as many people in need as possible (23). The information and communication technologies (ICTs), especially the Internet and mobile devices, offer important advantages for reaching different target groups (24). ICTs offer accessibility with an attractive cost-effective relationship for the challenges of mental disorders and prevention.

In the case of adolescents, these advantages have even greater potential because they are considered “*digital natives*.” Therefore, this population is not as hindered by potential obstacles and barriers, such as acceptability of technology, self-concordance, digital format, etc. Most adolescents are fully immersed in digital worlds, and their activities, relationships, and concerns are being defined by technologies.

In sum, ICTs can offer several advantages for fostering individual competencies, resources, and psychological strengths during adolescence. However, this area of research has hardly been considered. Thus, this paper seeks to contribute to this research gap and clarify the current state of the art. The paper starts with an explanation of the role of positive psychology (PP) in preventing mental health problems and promoting well-being and resilience in the adolescent population. Then, it presents an overview of the advantages of ICTs, specifically focusing on delivering mental health interventions for youth *via* the Internet. Finally, the results of a literature review combining the three fields of research—PP and Internet interventions in youth—are presented, providing a brief description of the systems developed so far and a summary of selected features of these studies.

### The Role of PP

Positive psychology is the study of the conditions and processes that contribute to the flourishing or optimal functioning of people, groups, and institutions (25). Its main areas of study can be grouped in four categories (26): (a) positive emotions (e.g., happiness, flow, etc.) and their effects on psychological and physical functioning; (b) positive individual traits (e.g., values, talents, etc.) and their protective role in different physical and psychological disorders; (c) positive interpersonal relationships (e.g., friendship, marriage, etc.); and (d) positive institutions (e.g., family, school, business, etc.).

Positive psychology has also been interested in the development of positive psychology interventions (PPIs). Sin and Lyubomirsky (27) defined them as intentional activities specifically addressed to cultivate positive feelings, cognition, and behaviors. PPIs are exercises (e.g., counting your blessings, practicing kindness, expressing gratitude, using personal strengths, etc.), which have demonstrated empirically to increase positive emotions, satisfaction with life, or other positive states. In this sense, PPIs are not activities focused on remedying or healing pathological or negative aspects, but on contributing to well-being and health through activities aimed to enhance positive affect, cognitions, and behaviors, and they may be considered as a complementary strategy in mental health promotion and treatment (28).

Currently, there is substantial evidence about their effectiveness in improving well-being, reducing depressive symptoms (27, 28), and increasing the effectiveness of available psychological treatments (29). PPIs have also emerged as promising tools in the promotion and prevention of mental health (30–33).

Due to the high prevalence of emotional problems in young people worldwide (7), skills for improving well-being and happiness should be taught to teenagers as well (34, 35). Adolescence is an optimal stage to do this because it is a crucial period in human personal and emotional development (identity construction, social relationships, etc.). PP tries to create a fresh conceptual framework that allows the development of interventions focused on human strengths and individuals’ potential, rather than on their deficits or the troubled image traditionally associated with adolescence (34). However, it is necessary to further investigate the development and application of PP interventions in young people because the evidence is still scarce (36–38). Research with PPIs has mostly been carried out with adult populations (28), and although some studies have shown the effectiveness of PPIs in increasing well-being in adolescents (39, 22), the need for more specific knowledge about the application of PPIs in the young population is evident (22).

### Technology and Interventions

The use of ICTs to enhance health services is increasing due to numerous advantages they offer to the health-care system. Increased accessibility to interventions, reliability (efficacy/effectiveness), and financial efficiency in health-care systems have been identified as relevant benefits of e-health in different studies (40–43). Its contributions to the five dimensions postulated by the RE-AIM model (reach, efficacy/effectiveness, adoption, implementation, and maintenance), a framework extensively used to assess the public health impact of interventions (44), are supported by a large body of literature. Moreover, in the case of adolescents, ICTs facilitate access to their habits, culture, communications, social connection, etc. (45, 46). A comprehensive report elaborated in 2010 detected that young people consume technological devices for at least the same amount of time an adult spends daily at his/her work place, they use ICTs 7 days a week, and several devices simultaneously (47). Therefore, using ICTs to approach adolescents could facilitate the implementation of preventive interventions. Recent studies highlight the advantages of technologies to engage this population, monitor their behaviors, and provide them with information, interventions, etc., although literature on effectiveness is limited (48–50).

### Positive Technology/Internet-Based Positive Technology

The brief duration of PPIs makes them good candidates for administration over the Internet, and online administration improves their dissemination. Proposals have been made to capture the fruitful relationship between PPIs and ICTs, and different terms have been suggested. Mitchell et al. (51) offered the term *online positive psychological intervention* and defended the potential of these interventions as an effective and sustainable health promotion tool within a comprehensive approach to mental health care. Riva et al. (52) and Botella et al. (53) proposed the term *Positive Technologies* to refer to the “scientific and applied approach that uses the technology for improving the quality of our personal experience with the goal of increasing wellness, and generating strengths and resilience in individuals, organizations, and society” [(53), pp. 1]. This field tries to combine and enhance the objectives of PP using all the possibilities offered by ICTs. Currently, there is already evidence of the effectiveness of these positive technologies in adult populations (28, 51), For example, Baños et al. (54) reported positive results of a self-guided Internet intervention to induce positive emotions and reinforce psychological resources. Shapira and Mongrain (55) also tested positively the effectiveness of two online exercises to help individuals experience self-compassion and optimism. Gander et al. (56) reported the impact of nine strengths-based positive interventions on well-being and depression in an Internet-based randomized placebo-controlled study, and these authors also offered data about the efficacy of PPIs *via* the Internet for older age groups (57). However, little work has been done in adolescence. Whereas there is a range of Internet-based well-being programs based on PP available to the general public (28, 51), there are not many Internet interventions specifically designed to promote well-being and resilience in adolescents and youth.

The present study conducts a narrative review that describes and discusses the state of the art of this field. Specifically, the purpose of this paper is to review the scientific literature on PPIs delivered through technological systems for promoting health and well-being in adolescent and youth populations.

## Materials and Methods

**Figure 1:**
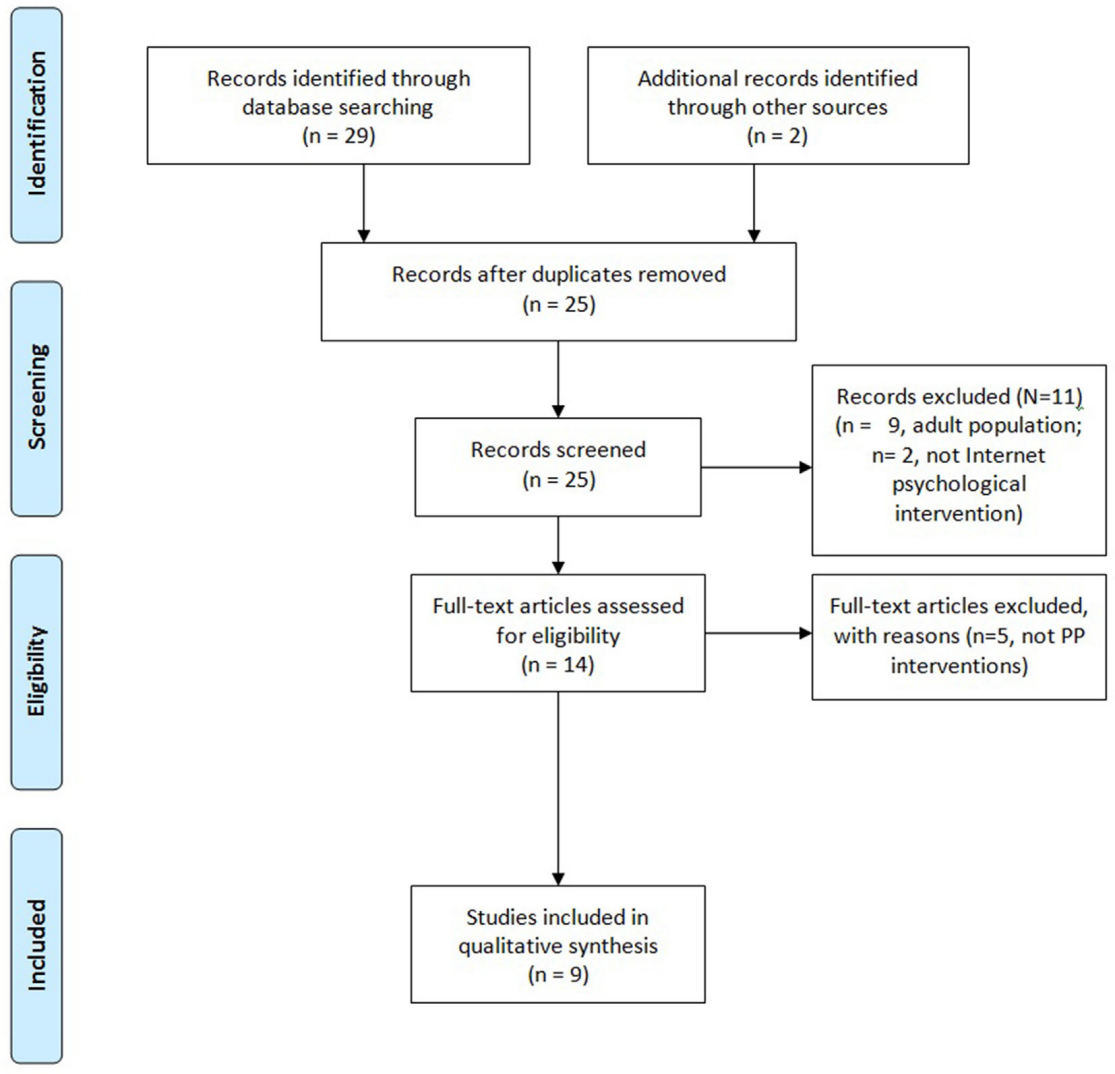
**Flow chart**.

In April 2016, a bibliographic search was conducted on the PsychINFO and PubMed databases. In addition, experts in the field were contacted for information on relevant material. Studies were eligible if: they have been focused on prove the efficacy of an online PPI to promote well-being and resilience in adolescent population; they were published until April 2016; they were English studies, and they were randomized controlled trials (RCTs). Studies were excluded if they were review articles. The search was limited to RCTs because a recent meta-analysis suggested that effectiveness of PPIs could be overestimated in not controlled studies (28). The key terms used were: “Positive Psychology” OR well-being OR wellbeing OR resilience OR happiness AND adolescent OR youth OR teenager AND “Internet intervention” OR online OR “Internet-based” OR “web-based” AND prevention OR “mental health.” Two independent evaluators reviewed and selected the studies. Those who were finally selected were reviewed by a third expert evaluator. Finally seven studies were identified about Internet-based PP programs for adolescents and youth. Consultation with international experts in the area identified one additional study (58), and one unpublished doctoral thesis (59) (Figure 1).

## Results

The literature review identified seven technological systems that include PPIs to promote well-being in adolescent and youth populations, which efficacy was tested in nine studies. All studies used a website to deliver the PPIs, one study also used social network (Facebook) and email (60), and another one also used text messages reminders (61). Regarding the efficacy of the interventions, in general all studies showed positive results, decreasing anxiety and depression scores, and increasing well-being.

A brief description of the systems and a summary of these studies are presented in Table 1.

**Table 1:** **Summary of findings**.

Intervention (reference)	System’s description	Technology used	Objective	Design	Sample	Outcomes
Bite Back (62)	It is aimed to promote mental health in young people during 6 weeks. It includes information and interactive activities related to nine domains: gratitude, optimism, flow, meaning, hope, mindfulness, character strengths, healthy lifestyle, and positive relationships	Website with individual login	To explore the feasibility of Bite Back to improve well-being and mental health outcomes in Australian youth	Randomized controlled trial (RCT) with two conditions: (1) Bite Back [interactive exercises and information across a variety of positive psychology (PP) domains] or (2) control websites (neutral entertainment-based websites that contained no psychology information)	Australian youth (*N* = 235): Bite Back (*n* = 120), control websites (*n* = 115)	Bite Back vs control condition: Bite Back participants with high levels of adherence: significant decreases in depression and stress and improvements in well-being Bite Back participants who visited the site more frequently: significant decreases in depression and anxiety and improvements in well-being

Bite Back (63)			To explore the feasibility of Bite Back at school	RCT with two conditions: (1) Bite Back (PP exercises and information) or (2) control condition (non-psychology entertainment websites)	Students from four high schools (*N* = 572): Bite Back (*n* = 313), control websites (*n* = 259)	Both conditions: reductions in depression, stress, and total symptom scores without any significant differences or significant improvements in life satisfaction scores post intervention.

InJoy (59)	It is organized in eight sessions to be used in school settings to prevent depression. It includes a set of positive psychology interventions (e.g., three good things, identifying signature strengths, mindfulness, gratitude letter, etc.), weekly self-reports, and a monitored discussion board	Website	To evaluate the effects of InJoy on reducing depressive symptoms in adolescents	RCT with two conditions: (1) InJoy or (2) control group, in high school settings	Adolescents (*N* = 58): intervention group (*n* = 26 freshman students), control group (*n* = 32 freshman students)	InJoy showed good but small effects on coping and emotion regulation, and less increase in the progression of depressive symptoms in students with low-risk of depression No significant effects on decreasing depressive symptoms in students at high risk of depression Significant differences in engagement compared to control group

InJoy (58)			It was conducted to evaluate engagement variables and to improve the effectiveness of system	Two studies: Study #1: addressed to identify areas where InJoy could be enhanced Study #2: evaluation of the enhanced version (InJoy revised)	Adolescents: Study 1: *N* = 162 freshman students Study 2: *N* = 170: intervention group (34 freshman students), control group (*n* = 36 freshman students)	Revised InJoy version was rated higher on the targeted social learning domains and as significantly more engaging (helpful, interesting, and fun)

E-health4Uth (60)	It is a tailored intervention to promote well-being and health behaviors in adolescents. In this intervention lasting one classroom session (45 min approx.), adolescents completed a self-report questionnaire over the Internet to assess health-risk behavior and well-being, and then they were presented with a message for each topic. Adolescents were encouraged to read more information on the topics through links to relevant websites. At the end, participants were invited to follow the Facebook page to find more information. Additionally, adolescents could check a box for a self-referral to the nurse or send an email to the nurse	Website, email, and Facebook page	To evaluate the effect of E-health4Uth on well-being and health behaviors	Cluster RCT with three conditions: (1) E-health4Uth, (2) E-health4Uth and consultation group (were subsequently referred to a school nurse for a consultation if they were at risk of mental health problems), or (3) control group (i.e., care as usual)	Third- and fourth-year secondary school students (*N* = 1,702 adolescents). School classes (clusters) were randomly assigned to: E-health4Uth group (*n* = 533), E-health4Uth and consultation group (*n* = 554), control group (*n* = 615)	Compared to the control group: E-health4Uth intervention showed minor positive results in health-related quality of life and condom use during intercourse among adolescents of Dutch ethnicity E-health4Uth and consultation intervention showed minor positive results in the mental health status of adolescents, but a negative effect on drug use in boys

Mother–Daughter Prevention Program (64)	It is a substance abuse prevention program for adolescents. The intervention consisted of nine online sessions (35-to-45-min). The program aims to strengthen the quality of girls’ relationships with their mothers while increasing girls’ resilience in resisting substance use. Each session includes interactive modules for girls and mothers to complete together	Website with interactive modules	To evaluate the effects of the program in the prevention of substance abuse in Asian American adolescent girls (until 2-year follow-up)	RCT with two conditions: (1) Mother–Daughter Prevention Program or (2) control group	Adolescent girls aged 10–14 and their mothers (*N* = 108): intervention arm (*n* = 56), test only control arm (*n* = 52)	Compared to the control group: Intervention-arm girls reported higher levels of mother–daughter closeness, greater mother–daughter communication, more maternal monitoring, and enhanced parental rules again substance use at 2-year follow-up. Intervention-arm girls also reported stronger self-efficacy, greater refusal skills, and lower intention of using substances in the future Furthermore, they reported significantly fewer instances of using alcohol, marijuana, and prescription drugs for non-medical purposes We did not detect a significant time × intervention effect on girls’ depressive mood, body esteem, and substance use normative beliefs

Transdiagnostic trait-focused online intervention “PLUS” (65)	It is an online intervention for students to help them learn more about their strengths and weaknesses and how to deal with the challenges of student life It consists of five modules addressing a range of CBT interventions. The modules were designed to help students recognize and reduce unhelpful behaviors and thoughts resulting from certain personality risk factors	Website with online modules	To evaluate the efficacy of the program in reducing symptoms of common mental disorders in university students	RCT with two conditions: (1) PLUS online intervention or (2) control intervention	Undergraduate and postgraduate students aged 18 or older (*N* = 1,047): PLUS online intervention (*n* = 519), control intervention (*n* = 528)	Compared to the control intervention: The trait-focused intervention reduced depression and anxiety scores in students at high risk Furthermore, self-esteem was improved No changes were observed in the use of alcohol or disordered eating

Out and online program (66)	It is an online intervention designed to reduce anxiety and depressive symptoms and enhance well-being in same-sex attracted young adults. It was developed as a stand-alone resource that should complement face-to-face therapy It consists of seven modules with mental health and well-being information and exercises, and the content was personalized for this specific population An additional module on prevention and help for suicidal thoughts is also available	Website with online modules	To examine the effectiveness of the program for reducing anxiety and depressive symptoms and improving well-being in SSAYA	RCT with two conditions: (1) online intervention or (2) waiting list control group	Same-sex attracted young adults with anxiety and/or depressive symptoms and mild to moderate psychological distress (aged between 18 and 25 years) (*N* = 200)	The work is in progress

Nothing ventured nothing gained (61)	It is an online adolescent and parenting intervention designed to improve physical and mental health outcomes (anxiety, depression, quality of life, well-being, self-efficacy, resilience, etc.) in adolescents with type 1 diabetes and their parents. The adolescent platform consists of five sessions to be completed over a 6-week period. The parent intervention has similar contents, but adapted to them	Website with individual login, mail, and text messages reminders	To examine the program’s effectiveness in improving adolescents’ mental health (depression and anxiety)	RCT with two conditions: (1) online intervention or (2) waiting list control group	Adolescents with type 1 diabetes (aged 13–18 years and one of their parents/guardians) (*N* = 120)	The work is in progress

## Discussion

Only seven ICT-based PPI for promoting health and well-being in adolescent and youth populations were identified in the literature review, and only nine studies testing their efficacy. Therefore, our first conclusion is that more controlled studies are needed. Furthermore, no longitudinal studies were identified, and these ICT-based prevention interventions should also prove their effectiveness through longitudinal studies that can clearly demonstrate whether the protective factors (virtues, strengths, etc.) are maintained in adulthood. Only one intervention (Mother–Daughter Prevention Program) reported data from a 2-year follow-up (64).

Another conclusion has to do with the design of the ICT-based PPIs. They should be designed considering the specific features of the target users. Both the contents (language, instructions, images, etc.) and other format and design elements are crucial in engaging teens. For example, InJoy was modified to improve its effectiveness and enhance its ability to engage. Compared to the initial version, the recent one was rated higher by students on the targeted social learning domains and found to be significantly more engaging (helpful, interesting, and fun) (58). Another example of adapting the systems to young people’s characteristics is the use of Internet social networks. E-health4Uth uses social networks (Facebook). The use of these resources could increase the attractiveness of these programs for young people and their adherence to them (47).

This review also identifies the importance of considering the contexts where the interventions will be delivered. For example, Bite Black was not more effective than the control task when used in schools, perhaps because teens perceived the application as an additional “school task” (63). This finding suggests the need to explore which technological systems and which PPIs best fit the different settings (school, family, leisure, etc.). Schools offer the possibility of reaching a large number of users, but the adaptation of interventions to increase their effectiveness should be considered.

Another relevant issue is the role of different technologies to deliver PPIs. Technological advances, especially related to smartphones, sensors, and virtual/augmented reality in daily life, could be very useful in promoting well-being. In our revision, it draws attention the limited number of ICTs used. It is important to explore this field because young people use ICTs every day through several mobile devices simultaneously (47) and this trend is expected that increases in the coming years. Currently, smartphones are main channels of communication, socialization, and entertainment for adolescents, and therefore to use them as devices to deliver PPIs could be appropriate. In this review, no system uses mobile phone resources to carry out the interventions and nurture interpersonal relationships. However, since smartphones or similars were not considered as keywords it is possible that some systems were not detected. This is a limitation in our search that should be considered.

This paper focuses on the benefits of using ICT-based PPIs to promote wellbeing in adolescents and youth. However, it should be highlighted that these interventions could also have some negative effects and potential dangers, if misused. It is necessary to be cautious when designing a prevention program, as people with more severe mental disorders might consider these programs to be substitutes to psychological treatments. In addition, other important aspects should also be taken into account in the e-mental health field (67), including confidentiality, privacy, and rigor of contents. Further work needs to consider all these issues, because despite limitations and potential dangers, ICT-based PPIs offer new mental health opportunities, especially for adolescents, and important benefits in terms of sustainability and accessibility. This review shows that some controlled studies have already been conducted, but much more research is required to establish the efficacy of these preventive interventions, including long-term follow-up studies. Successful prevention of mental disorders and mental health promotion are priorities worldwide, and ICTs can be a great ally, especially among young people.

## Author Contributions

All authors (RB, EK, AM, GR, AG, and CB) jointly contributed to write the manuscript and all of them approved the final version for submission. Literature search has been done by EK and AM. Studies selected were reviewed by a third expert evaluator (RB). All authors contribute to the discussion of the literature review.

## Conflict of Interest Statement

The authors declare that the research was conducted in the absence of any commercial or financial relationships that could be construed as a potential conflict of interest.
